# To stay or leave: an integrative review of factors, personas, and recommendations for retaining family physicians in Canada

**DOI:** 10.1186/s12875-025-03128-x

**Published:** 2025-12-01

**Authors:** Udoka Okpalauwaekwe, Brian K. MacPhee, Lindsay Balezantis, Vivian R. Ramsden, Angela Baerwald

**Affiliations:** 1 Research Division, Department of Family Medicine, College of Medicine, University of Saskatchewan, Saskatoon, SK S7M 3Y5 Canada; 2 College of Medicine, University of Saskatchewan, Saskatoon, SK S7M 3Y5 Canada; 3 Research Division, Department of Family Medicine, College of Medicine, University of Saskatchewan, Saskatoon, SK S7M 3Y5 Canada; 4 Women’s Health Research Laboratory, Department of Family Medicine, College of Medicine, University of Saskatchewan, Saskatoon, SK S7M 3Y5 Canada

**Keywords:** Family physicians, Physician retention, Canada, Rural medicine, Primary care, International medical graduate, Community integration, Workforce planning

## Abstract

**Introduction:**

Family physicians are the cornerstone of primary health care in Canada. Yet, retention remains a growing concern. Challenges in retaining family physicians poses serious implications for healthcare accessibility, continuity, and equity across all practice contexts in Canada, including (but not limited to) rural, remote, urban, and underserved communities. Currently, 25% of Canadians do not have a primary care provider. While much attention has been given to recruitment, less is known about the multifaceted and intersecting factors that influence whether practicing family physicians and family medicine trainees (including Canadian Medical Graduates (CMGs) and International Medical Graduates (IMGs)), remain in sustained comprehensive practice in Canada. This review synthesizes the literature to identify key drivers of family physician retention and offers evidence-based recommendations.

**Methods:**

We conducted an integrative review of peer-reviewed literature published between January 1, 2000, and March 30, 2025, following Whittemore and Knafl’s five-stage methodology. A systematic search was carried out across five electronic databases. Included studies were assessed for quality and thematically analyzed using a five-domain coding framework: personal, family, community, professional, and structural/systemic. Composite personas were developed to illustrate recurring physician retention trajectories and evidence-based recommendations were thematized across our five-domain coding framework.

**Results:**

Of the 1,613 records screened, 23 studies met inclusion criteria. Factors influencing retention were identified across all five domains. Structural and professional barriers, including licensure restrictions, administrative burden, and limited autonomy, emerged as the most consistent deterrents. Facilitators included strong community ties, spousal support, team-based practice environments, and access to continuing professional development. We identified and developed seven physician personas to create a portrait of the diverse experiences of family physicians in Canada. Key recommendations included reforming licensure and payment models, enhancing mentorship and CME access, supporting spousal integration, and fostering culturally safe, community-rooted team-based practice models.

**Conclusion:**

Retaining family physicians in Canada is a relational challenge that requires collaborative, multi-level change. Tailored, context-specific retention strategies co-designed with physicians and communities can enhance sustainability and health equity especially in rural, remote and underserved communities.

**Supplementary Information:**

The online version contains supplementary material available at 10.1186/s12875-025-03128-x.

## Introduction

Canada’s healthcare system operates under a publicly funded, universally accessible model, where primary care services are predominantly delivered by family physicians (FPs) who function as independent practitioners [1]. While they are not employed by the government, family physicians are remunerated primarily through publicly administered provincial or territorial health plans, most commonly via fee-for-service (FFS) models; however, alternate and blended payment models are increasingly used [2]. Nurse Practitioners (NPs) and other regulated health professionals (e.g. pharmacists) also provide important primary care services, particularly in rural, remote, and team-based care settings [3, 4]. Despite the central role of FPs in Canada’s healthcare systems, there remains growing challenges related to recruitment and especially retention, straining Canada’s family physician workforce [5, 6]. These challenges are deeply intertwined with how care is organized, delivered, and supported within this publicly funded model.

Retention of FPs in Canada remains a persistent and complex challenge, with serious implications for healthcare accessibility, continuity, and equity. The impacts of family physician shortages are particularly noticeable in rural, remote, and underserved communities (these include inner city areas with provider shortages, Indigenous communities, and urban or rural areas with high social vulnerability and communities with systemic barriers to care) [5–7]. Despite intensified recruitment efforts, the ability to retain both Canadian Medical Graduates (CMGs, physicians who completed their medical training in Canada), and International Medical Graduates (IMGs, physicians who obtained their medical degrees outside of Canada) in comprehensive family practices continues to pose challenges, often contributing to recurrent physician turnover and disrupted care continuity [5, 7]. Recent reports from the College of Family Physicians of Canada (CFPC) estimated that more than 6.5 million Canadians were without a regular family physician; at the same time, nearly one in five family doctors were considering reducing or leaving comprehensive practice altogether [6]. This widening gap in access to primary care was further underscored by the Canadian Medical Association’s (CMA) 2023 Physician Workforce Survey which reported rising burnout among family physicians and a declining interest in family medicine as a long-term career–both contributing to physician migration, particularly among early-career and rural physicians [8]. Additionally, in 2022 the Canadian Institute for Health Information (CIHI) documented that the effects of poor physician retention were disproportionately borne by Indigenous communities and structurally marginalized populations, where disruptions to care continuity could exacerbate already existing health inequities [9].

Since 2000, numerous strategies have been implemented in various Canadian provinces to attract physicians to family medicine and rural practice [7]. While some initiatives (e.g., enhanced rural training tracks, community-based onboarding, and team-based care models) have shown success in improving retention in rural and underserved areas [10, 11], others, such as return-of-service agreements and one-size-fits-all licensure pathways, have often fallen short due to limited alignment with physician goals and inflexibility across career stages or geographic regions [12–14]. A core limitation in workforce planning has been the tendency to approach retention as a linear, one-size-fits-all issue; overlooking the complex and evolving interplay of personal, family, professional, systemic, and community-level factors that shape physicians’ decisions to stay or leave [6, 7, 15, 16]. Notable challenges include administrative burden [13], excessive workloads [17], limited autonomy [7], and strained work-life balance [18], as well as issues related to training background, lack of team-based care, and how well physicians fit into the communities they serve (i.e., community integration) [7, 19–21]. While CMGs and IMGs share some of these challenges, IMGs may face particularly unique challenges linked to licensing processes [13], practice integration [7], social and cultural isolation, and experiences of systemic exclusion (including racism, xenophobia and limited institutional supports for equitable workforce integration) [7, 22, 23].

Recognizing the complex dynamics of providing primary care in Canada, we undertook this integrative review to offer a comprehensive understanding of the factors influencing family physician retention. Rather than limiting the lens to one region, career stage, or training pathway, this review synthesizes evidence across geographies and physician backgrounds to identify shared and divergent drivers of retention. Building on prior work focused primarily on IMGs [7], this study incorporates both CMG and IMG perspectives from across Canada’s provinces and territories. Through this synthesis, we developed simulated physician personas grounded in real-world retention narratives to illustrate diverse career trajectories and inform context-specific recommendations. Ultimately, this review provides an integrated lens on why physicians stay or leave practice, and identifies key leverage points for sustainable workforce reform.

## Study objectives

Our study uses an integrative review methodology to explore the retention of family physicians across Canada’s primary care landscape, encompassing varied practice locations (urban, rural, remote), settings (office-based, hospital, emergency departments, long-term care, etc.), and scopes (generalist or focused practice areas such as obstetrics or MAID, etc.). Retention within this context refers to physicians’ sustained engagement within family medicine practice (regardless of setting such as clinic, hospital, emergency, or home care) rather than migration into other specialties or out of clinical practice. Our integrative review was guided by the following objectives:

- To identify and synthesize personal, family, professional, community, and systemic factors that influence the retention or attrition of family physicians (both CMGs and IMGs) within Canadian locations between 2000 and 2025.
- To examine how personal, family, professional, community, and systemic factors interact across domains and over time, shaping physicians’ decisions to stay or leave family practice, particularly in rural, remote, and underserved areas.
- To identify and develop conceptual physician personas that illustrate patterns of experience, motivation, and vulnerability related to retention.
- To map evidence-informed recommendations to specific personas and contexts, offering practical strategies for improving retention across varied physician backgrounds and practice environments.

## Methodology and methods

### Study design

An Integrative Review design was employed to systematically synthesize diverse forms of evidence (i.e., quantitative, qualitative, and mixed methods) related to family physician retention in Canada between January 01, 2000, and March 30, 2025. Integrative reviews allowed for the synthesis of diverse types of evidence (quantitative, qualitative, theoretical, and empirical) to provide a more comprehensive understanding of complex phenomena [24, 25]. Unlike Systematic Reviews, which typically focus on a narrowly defined question using primarily empirical studies [26], or Scoping Reviews, which aim to map the breadth and extent of literature on a topic without deep synthesis [27, 28], Integrative Reviews go further by critically analyzing, comparing, and conceptually integrating findings from multiple methodologies and sources; applying broad and flexible approaches [24, 25]. We chose an integrative approach to go beyond description and mapping, allowing for conceptual synthesis, identification of cross-domain interactions, and the development of personas that illustrate distinct patterns of retention challenges and opportunities among family physicians in Canada (including CMGs and IMGs).

We adhered to the Whittemore and Knafl’s [25] methodological framework for Integrative Reviews (which includes problem identification, literature search, data evaluation, data analysis, and data presentation) using it as our guiding methodological framework. We reported our findings using the PRISMA-ScR (Preferred Reporting Items for Systematic Reviews and Meta-Analyses extension for Scoping Reviews) [28] checklist to promote transparent and comprehensive reporting (see Supplementary File A). While our review followed an integrative design, we selected PRISMA-ScR [28] because there are no standardized reporting guidelines for integrative reviews, and the PRISMA-ScR has been widely applied across diverse review methodologies due to its clarity, adaptability, and breadth.

### Protocol and registration

No protocol registration was required prior to the commencement of this study.

### Eligibility criteria

Studies were included if they met the following criteria:

- *Population*: Focused on Canadian family physicians (FPs) practicing in Canada.
- *Place of Study*: Conducted in any province or territory in Canada.
- *Geography*: Covered any setting in Canada including urban, metropolitan, rural, and remote areas.
- *Healthcare Area*: Addressed physician retention (i.e., decisions to leave or remain) across different scopes of practice (e.g., comprehensive care, focused areas such as obstetrics or MAID, etc.), settings (e.g., community clinics, emergency departments, hospitals, long-term care, or home-based practice), and geographic contexts.
- *Language*: Published in English.
- *Time Period*: Published between January 1, 2000, and March 30, 2025.
- *Study Types*: Included original empirical studies (qualitative, quantitative, and mixed-methods) and review articles that synthesized empirical data (e.g., integrative reviews, systematic reviews, meta-analyses, mixed-method syntheses, policy analyses, etc.).

Studies were excluded if they:

- Focused on populations other than family physicians (e.g., specialists, nurse practitioners, nurses).
- Were conducted outside of Canada.
- Focused on non-primary care settings (e.g., medical education or secondary/tertiary care).
- Were published in languages other than English.
- Were published prior to 2000.
- Were theoretical or conceptual in nature, or published as protocols, editorials, commentaries, opinion pieces, case reports, dissertations, conference abstracts, or other grey literature.

Table 1 provides a side-by-side summary of the inclusion and exclusion criteria for further reference. We chose the year 2000 as the starting point for this review to align with a period of significant health workforce reform in Canada [2], including the early implementation of national retention and recruitment strategies targeting rural and family medicine (e.g., return-of-service programs, rural locum schemes, and primary care renewal initiatives) [29, 30]. This time frame also reflects when the issue of physician retention began receiving broader policy and scholarly attention in Canada [29].

**Table 1:** Eligibility criteria for review study

Criteria	Inclusion	Exclusion
Population	Canadian Family Physicians	Other health professionals such as specialists, nurse practitioners, nurses, etc.
Place of study	Canada	Any country other than Canada
Geography	All areas including urban, metro urban, cities, and rural	All areas outside of Canada
Healthcare areas	Physician retention, including decisions to leave or remain across different scopes of practice (e.g., comprehensive, focused care such as obstetrics or MAID, etc.), settings (e.g., office, hospital, ED, LTC), and geographic locations (urban, rural, remote) within Canadian primary care.	Secondary or tertiary care, medical education, medical training
Language	English	Non-English
Time period	2000–2025	< 2000
Study type	Original studies and other synthesis material (reviews, policy analysis, meta-analysis, mixed method synthesis, data synthesis, etc.) published in a peer-reviewed journal	Theoretical or conceptual papers, protocols, editorials, commentaries, opinion pieces, dissertations, conference abstracts, case reports, historical and grey literature.

### Information sources and search strategy

We collaborated with an academic librarian at the University of Saskatchewan to design a comprehensive search strategy that identified relevant groups of terms. Key search terms were organized into three conceptual groups: “family physician” (and synonyms), “Retention” (and synonyms), geography (and synonyms) and “Canada”. The following electronic databases were searched: MEDLINE (Ovid), PubMed, Scopus, Web of Science and ERIC (Education Resource Information Center). Searches were conducted for articles published from January 1, 2000, to March 30, 2025. Reference lists of included articles were also hand-searched for additional sources. The full search syntax used for each database can be found in Table 2.

**Table 2:** Keyword search syntax for library database queries

1. Population/
2. Family adj1 physicians* OR general adj1 practitioner$ OR nurse adj1 practitioner* OR International adj1 medical adj1 graduate* OR Foreign adj1 medical adj1 graduate OR Migrant adj1 physician* OR oversea* adj1 trained adj1 physician* OR oversea* adj1 trained adj1 health adj1 professional.ti.ab*
3. Retention
4. Physician adj3 retention OR stay OR exit OR turnover OR attrition OR job adj2 retention OR job adj2 satisfaction OR fulfilment OR career adj2 advancement OR contentment OR workforce stability OR job adj2 dissatisfaction.ti.ab
5. Practice geography/
6. Urban OR Metro* OR Rural adj2 medicin* OR rural adj1 population* OR rural adj1 communit* OR rural adj1 practice OR rural adj2 practice or rural adj2 health OR rural adj2 health adj1 servic*.ti.ab
7. Location/
8. Canada OR Alberta OR British adj1 Columbia OR Manitoba OR New adj1 Brunswick OR Newfoundland adj1 and abj1 Labrador OR Northwest adj1 Territor* OR Nova adj1 Scotia OR Nunavut OR Ontario OR Prince adj1 Edward adj1 Island OR Quebec OR Saskatchewan OR Yukon.ti.ab
9. #2 AND #4 AND #6 AND #8

### Selection of sources of evidence

**Fig. 1:**
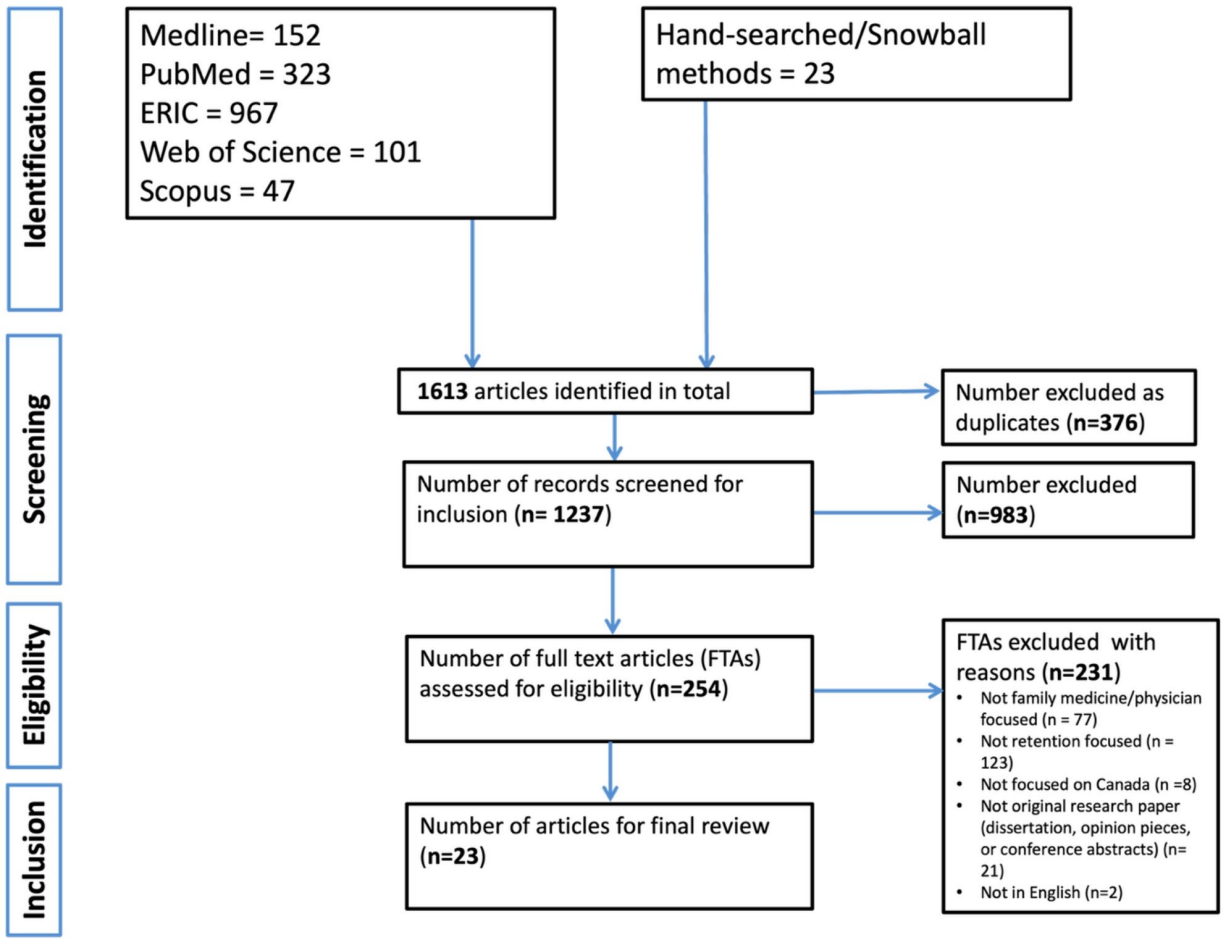
PRISMA flowchart showing selection of articles for integrative review

All identified records were imported into Covidence–a web-based collaboration software platform that streamlines the production of systematic and other literature reviews (Veritas Health Innovation, Melbourne, Australia. Available at www.covidence.org). Thereafter, duplicate records were removed. We applied two iterative stages to select articles for this review. They were: (1) title and abstract screening, using our predefined eligibility criteria; and (2) full-text article (FTA) review, where eligibility was further confirmed or rejected with documented reasons. Two reviewers (UO and BKM) independently reviewed in both stages and where we had conflicts, resolved them through consensus or arbitration by a third reviewer (LB). The selection process was summarized in the PRISMA-ScR Flow Diagram in Fig. 1.

### Data charting and extraction

We created a structured data extraction template within Covidence to capture and extract data under the following title fields: article title, citation and study objective; study type, study design, location, and setting; physician population focus (CMGs, IMGs, or both); main retention-related findings categorised as barriers and/or facilitators; and recommendations/policy implications. To meaningfully capture the diverse influences (i.e. barriers and facilitators) on physician retention, we drew on a pre-developed five-domain coding framework personal, family, community, professional, and structural/systemic. These domains have been informed in previous studies on workforce retention [7, 15, 16] and reflect the complex, relational nature of retention decisions across career stages and practice settings. We chose to use this framework to enable a holistic synthesis of findings, moving beyond isolated factors toward a more integrated understanding of the lived experiences of family physicians in Canada. All data extracted were then exported into Microsoft Excel (Microsoft Corporation, Version 16) for cleaning, coding, and data synthesis.

## Data analysis and synthesis

### Barriers and facilitators of physician retention

A thematic analysis was undertaken using a deductive coding approach, guided by a predefined framework comprising five interrelated domains that have been consistently identified in the literature as influencing physician retention in Canada: personal, family, community, professional, and structural/systemic [7, 15, 16]. Each study’s findings were coded into these domains to facilitate thematic synthesis and the development of barrier–facilitator matrices. Where relevant, cross-domain influences were acknowledged through dual coding, allowing us to organize findings while maintaining fidelity to the complexity of physician retention narratives across settings and populations.

### Persona development

To enhance interpretability and support knowledge translation, we used patterns identified across the five domains to develop composite physician “personas.” These personas were not drawn from individual studies but synthesized from recurring narrative patterns. They were designed to offer relatable, human-centered profiles that decision-makers could use to better understand the complex and varied contexts of physician retention. While not exhaustive, the personas reflected the diverse migration pathways, motivations to stay or leave, and the vulnerabilities of Canadian family physicians practicing in various areas. These personas served in this study, as narrative tools to help interpret the findings, uncover systemic gaps, and inform the development of context-specific retention strategies. We constructed these conceptual personas by: (a) identifying clusters of co-occurring themes (e.g., burnout + isolation + spousal dissatisfaction); then (b) mapping them to demographic and professional traits (e.g., IMG status, early career stage, rural exposure); and finally, (c) synthesizing them into narrative personas, by including motivators, barriers, likely outcomes, and/or targeted policy levers. These personas were validated to ensure credibility and relevance through both informal (personal and group discussions) and formal discussions with practicing family physicians across urban, rural, and remote settings in Saskatchewan and other provinces in Canada (British Columbia, Ontario, Nova Scotia and Manitoba) whom the first author (UO) had interactions with at conferences, symposiums, and meetings where early findings of this work were presented. We also sought feedback from these engagements to iteratively refine the personas, ensuring consistency with observed patterns across the data sources (enhancing their applicability through informal triangulation and content validation).

### Recommendations to enhance physician retention

A thematic analysis of data extracted from included studies on recommendations to enhance physician retention in Canada was conducted and data were mapped with our coding framework across the five domains (i.e., personal, family, community, professional, and systemic).

### Quality assurance and critical appraisal of included studies

Using the Joanna Briggs Institute (JBI) Critical Appraisal Toolkit [31], we evaluated the methodological quality of each included article. Two reviewers (UO and BKM) independently assessed the studies, and any discrepancies were resolved through discussion and consensus. A summary of the critical appraisal process and results is provided in Supplementary File B. While no studies were excluded based on appraisal results, this process provided added confidence in the methodological quality and interpretive value of the included evidence.

### Reflexivity and trustworthiness of study

UO is an IMG and participatory primary care researcher with the Department of Family Medicine, College of Medicine, University of Saskatchewan; BKM and LB are medical trainees (undergraduate health sciences student and family medical resident respectively) in the College of Medicine, University of Saskatchewan; VRR is a participatory primary care researcher and Director of Research with the Department of Family Medicine, College of Medicine, University of Saskatchewan; and AB is a practicing family physician, and clinical researcher with the Department of Family Medicine, College of Medicine, University of Saskatchewan. As a team, we met regularly to collaboratively interpret, validate, and refine the findings, ensuring we were providing a synthesis that was informed by the diversity of perspectives in the literature and enriched by insights from practicing family physicians and primary care researchers. Additionally, during these debriefing meetings, we critically reflected on how our respective roles and affiliations might have introduced interpretive biases into the analyses/findings.

## Results

### Selection of sources of evidence

Our initial search across five electronic databases and hand-searching yielded a total of 1,613 records. After removing 376 duplicates, 1237 articles were screened based on titles and abstracts. Of these, 254 full-text articles (FTAs) were reviewed in detail. Following FTA screening, 23 studies were included in the final synthesis. The most common reasons for exclusion at the full-text review stage included more focus on recruitment rather than retention, studies not addressing family physicians specifically, lack of relevance to the Canadian context, and non-original research (e.g., protocols, dissertations, conference abstracts, etc.). We illustrated the process of article identification, selection and inclusion in the PRISMA Flow Diagram in Fig. 1.

### Characteristics of sources of evidence

The included studies employed a diverse range of methodologies: 11 (47.8%) were qualitative, 9 (39.1%) were quantitative, and 3 (13%) used mixed methods. Most studies (65.2%) were published between 2010 and 2019. With regards to geographical scope, 9 of 23 studies (39.1%) had a national or multi-provincial focus, while the remainder concentrated on specific provinces including Alberta (4/23; 17.4%), Newfoundland and Labrador (4/23; 17.4%), Ontario (4/23; 17.4%), Saskatchewan (3/23; 13%), Quebec (3/23; 13%), Nova Scotia (2/23; 8.7%), and Manitoba (2/23; 8.7%). These studies covered a range of settings including urban, rural, and remote practice environments. While three studies (13%) focused exclusively on either IMGs or CMGs, the majority (17/23; 73.9%) included both groups. A summary of the methodological and descriptive characteristics of the included studies can be found in Table 3.

**Table 3:** General and methodological descriptive characteristics of included studies (*n* = 23)

Publication year	*n* (%)	Article citation
2000–2009	4 (17.4)	[13, 32–34]
2010–2019	15 (65.2)	[10, 12, 17, 22, 35–42]
2020–2025	4 (17.4)	[43–46]
Provinces*		
AB	4 (17.4)	[12, 14, 15, 45]
BC	2 (8.7)	[44, 46]
MB	2 (8.7)	[39, 41]
NFL	4 (17.4)	[13, 33, 36, 40]
NS	2 (8.7)	[44, 46]
ON	4 (17.4)	[37, 38, 44, 46]
SK	3 (13.0)	[10, 17, 36]
QC	3 (13.0)	[37, 42, 43]
Pan-Canadian/Multiple provinces	9 (39.1)	[18, 22, 32, 34, 35, 37, 38, 44, 46]
Family Physician category		
CMGs focused	3 (13.0)	[34, 36, 43]
IMG focused	3 (13.0)	[32, 39, 44]
Both IMGs and CMGs	17 (73.9)	[10, 12–15, 17, 18, 22, 33, 35, 37, 38, 40–42, 45, 46]
Region		
Urban	0 (0.0)	--
Rural	13 (56.5)	[12, 14, 15, 17, 18, 32, 34–36, 40, 43–45]
Both	10 (43.5)	[10, 13, 22, 33, 37–39, 41, 42, 46]
Study type		
Quantitative	9 (39.1)	[13, 22, 33–35, 37–39, 41]
Qualitative	11 (47.8)	[10, 12, 15, 32, 36, 40, 42–46]
Mixed Methods Research	3 (13.0)	[14, 17, 18]
Data collection type		
Primary data	16 (69.6)	[10, 12, 14, 15, 17, 18, 34–36, 39, 41–46]
Secondary data (Data Registry/reviews/admin data)	7 (30.4)	[13, 22, 32, 33, 37, 38, 40]

*Multiple overlap for cited studies

### Results of individual sources of evidence

A summary of the unique sources of evidence for this study can be found in Table 4. All studies included in the final synthesis met the eligibility criteria outlined in the Methods and Table 1. Table 4 summarizes how each included study contributed evidence relevant to one or more of our research objectives.

**Table 4:** Characteristics of included studies (*n* = 23)

Author (citation)	Study Objective	Study type, Design, Location, Setting, and Physician focus	Relevant Findings		Practical implications and indications to enhance workforce retention
			Barriers	Facilitators	
Ampofo-Addo et al. 2016 [17].	To understand factors that influence location decision of family physicians in Saskatchewan (SK), Canada	Study type: mixed methods research Design: Surveys and Interviews Location: Saskatchewan, Canada. Setting: Rural Physician focus: IMGs and Canadian Medical Graduates (CMGs).	• Family: lack of spousal and family supports. • Community: Lack of community support, community disintegration • Professional: Burnout, high call rotation, few staff, lack of team support and educational opportunities • Structural/systematic: Non-sustainable model of care delivery, poor infrastructure/working conditions	• Personal: preference for rural life, friendly climate and rural activities. • Family: Spousal employment/family support, access to children’s education • Community: social integration • Professional: Good workload, collegiality, scope of work and financial compensation	• Match physicians with communities that align with their family, work-life, and lifestyle expectations. • Provide financial incentives only to communities that cannot meet those requirements. • Foster collaboration among small communities for viable group practices
Asghari et al., 2017 [44].	To identify factors influencing decisions to work in rural or remote areas.	Study type: Qualitative Design: Interviews with rural physicians. Location: Quebec, Canada. Setting: Rural Physician focus: CMGs (implied).	• Personal: lack of extracurricular activities. • Family: lack of spousal employment and children’s education. • Community: remoteness, cultural or language barriers, and absence of spiritual or cultural centers. • Professional: difficulty accessing continuing education and specialists, burnout from high turnover and long hours, social challenges like maintaining a social life and anonymity. Poor collegial support and treatment from urban physicians.	• Personal: preference for rural life, previous education or life experience in rural area • Family: spousal support to settle rurally, access employment opportunities. • Community: community appreciation, friendliness, security, privacy, intimacy. • Professional: wide scope of practice, access to CME, collegiality, positive working environment, and strong practice team • Structural: additional financial incentives to stay, and reimbursement for travels outside rural area	• Providing opportunities for professional development, creating a supportive work environment, enhancing collegial and personal support systems, • Ensuring a healthy work-life balance, providing adequate education for children, offering employment opportunities for spouses, and fostering community integration. Supporting and creating specific competencies targeting rural skills and supportive training environment.
Bosco et al., 2024 [18].	To understand factors influencing physicians’ decisions to leave/stay in rural practice and assess perspectives on national licensure	Study type: mixed methods Design: Environmental scan of 25 reports + qualitative and quantitative analysis of 2 national physician surveys (SRPC and CMA) Location: national focus Canada. Setting: Rural Physician focus: Both CMGs and IMGs	• Family: Family dissatisfaction • Community: limited community integration. • Professional: burdensome licensure requirements and fees for cross-provincial work. Poor work-life balance and burnout • Structural: Lack of infrastructure and locum coverage in rural areas.	• Community: community integration and team-based practice support. • Professional: National licensure enabling interprovincial mobility. Locum flexibility for semi-retired/retired physicians. Ability to access educational and locum opportunities across provinces.	• Implement national (pan-Canadian) licensure to reduce administrative barriers • Improve locum availability and infrastructure in rural areas. • Create turnkey, team-based rural practices. • Address family and community integration needs
Cameron et al., 2010 [12].	To explore community factors that promote physician retention in rural Alberta communities	Study type: Qualitative Design: Interview, Collective case-study design. Location: Alberta, Canada. Setting: Rural Physician focus: Both CMGs and IMGs	• None identified in study	• Community: Community appreciation (thank-yous, gifts, verbal praise). Personal and family connection to the community. Active support (advocacy, welcoming efforts, local fundraising). Appealing physical/recreational infrastructure (parks, activities). Mutual respect and reciprocity between community and physicians	• Strengthen community-physician relationships, • Involve communities in physician support, both socially and professionally. • Consider community-based interventions (e.g., welcome programs, community appreciation). • Promote reciprocal contributions between physicians and communities
Cameron et al. 2012 [15].	To explore professional, personal, and community factors influencing physician retention in rural Alberta.	Study type: Qualitative Design: Interview, Collective case-study design. Location: Alberta, Canada. Setting: Rural Physician focus: Both CMGs and IMGs	• Personal: lack of sufficient recreational assets, dissatisfaction of a physician’s spouse with the community. • Community: supply leading to burnout. Lack of community support • Professional: inadequate physician Preserving status quo. Lack of innovative drive from management	• Personal: goodness-of-fit, individual choice. • Family: spousal and family support. • Community: appreciation, connection, active support, physical and recreational assets, reciprocity • Professional: physician supply, dynamics, scope of practice, practice set-up, innovation, management and support. Advanced innovation and management support	• Considering the interplay between professional, personal, and community factors, • Linking recruitment with retention efforts, • Fostering positive doctor-patient relationships, promoting strong communities, and encouraging collaborative retention strategies led by both physicians and communities.
Chan et al., 2005 [34].	To examine influences on rural practice entry among physicians with different backgrounds.	Study type: Quantitative Design: Cross-sectional survey. Location: National scope, Canada. Setting: Rural. Physician focus: CMGs.	• Family: the influence of spouses’ preferences and proximity to family. • Structural: limited effectiveness of financial incentives for long-term retention, the small number of rural students entering medical school. Financial incentives are less effective for long-term retention.	• Personal: enjoyment of a rural lifestyle, and rural education during medical training • Family: Spouses’ preferences and proximity to family,	• Encouraging rural applicants to apply for medicine, • Making exposure to rural practice available during training, • Promoting the challenges and lifestyle of rural practice, and • Targeting both urban and rural students for recruitment and retention in rural areas.
Chauban et al., 2010 [43].	To assess incentives, satisfaction, future migration plans, and retention strategies among rural physicians in Canada	Study type: Quantitative Design: National cross-sectional survey. Location: National scope, Canada. Setting: Rural. Physician focus: IMGs and CMGs.	• Family: Inadequate family support or child education options • Professional: Lack of locum coverage (60% dissatisfaction). Poor professional backup (43%). Poor satisfaction with relief and on-call workload.	• Personal: Liking rural lifestyle (82%). Match with career interests (75%). Positive rural training experiences • Community: sense of community appreciation (84%) and belonging (76%). • Professional: Full scope of practice (84%).	• Improve access to locums and professional relief. • Maintain and promote full scope of practice in rural settings. • Invest in structured rural training experiences. • Address family and community integration. • Support intra-professional collaboration and use of technology for specialist support.
Dove N. 2009 [38].	To assess the potential of IMGs in addressing rural physician shortages in Canada.	Study type: Review/Policy Analysis Design: Policy perspective analysis and narrative critical discourse Location: Pan-Canadian and rural focused Setting: Rural Physician focus: IMGs only.	• Personal: Preference for urban migration. • Family: Limited spousal integration, job opportunities, and educational support for families. • Community: Lack of engagement, social and cultural isolation, discrimination, and high turnover. • Professional: Heavy workload, burnout, limited mobility, restricted practice scope, low compensation, and lack of training support. • Structural: Poor infrastructure, inconsistent licensing policies, short retention periods, and lack of national coordination in IMG recruitment.	• Personal: Attraction to rural life, previous rural training, and community appreciation. • Family: Spousal employment, family integration, and educational opportunities. • Community: Strong local support, cultural/religious networks, and diverse environments. • Professional: Balanced workload, career growth opportunities, full licensure, financial incentives, and access to continuing education. • Structural: Institutional support, clear contracts, well-equipped facilities, coordinated licensing, and ethical recruitment policies.	• Integrate IMGs into long-term workforce planning. • Enhance spousal and family support. • Focus on career development, community integration, and systemic workforce strategies beyond licensing. • Ensure access to mentorship, training, and career pathways. • Improve financial incentives, relocation support, and flexible payment models. • Adopt a national approach to IMG recruitment, licensing, and retention to improve workforce stability.
Grudniewicz et al., 2023 [46].	To identify the key factors that influence the practice choices of early-career family physicians in Canada	Study type: Qualitative Design: Framework Analysis using semi-structured interviews Location: BC, Ontario and Nova Scotia, Canada. Setting: Rural and Urban Physician focus: IMGs and Canadian Medical Graduates (CMGs).	• Family: personal responsibilities (e.g., childcare). • Professional: Lack of flexibility in practice models. Lack of mentorship or poor training experiences; overwork; limitations in payment models or scope	• Community: community connection; group practices; affiliation with other health professionals • Professional: positive training experiences; mentorship; supportive team environments; flexibility in practice schedule.	• Align training with desired practice models. • improve policy and payment systems to support flexibility and comprehensiveness. • Support family responsibilities and work-life balance. • Recognize diverse personal and professional goals
Levesque et al., 2018 [42].	To explore factors influencing physician retention in Quebec, Canada	Study type: Qualitative Design: Semi-structured interviews. Location: Quebec, Canada. Setting: Rural Physician focus: IMGs and CMGs.	• Professional: Burnout, limited mobility, or scope of practice • Structural: issues with continuity of care.	• Family: Spousal employment, family integration, opportunities for children • Professional: Opportunities to advance career, physician wellness, and work environment, large scope of practice, autonomy, and care continuity.	• Focusing on improving the work environment and professional life quality through Regional Medical Campuses (RMCs), which contribute to regional development, healthcare quality, and economic impacts. • Creating teaching opportunities.
Mathews et al., 2007 [33].	To assess whether IMGs trained at Memorial University of Newfoundland (MUN) are as likely as MUN and other Canadian medical graduates to work in Canada and Newfoundland and Labrador (NL)	Study type: Quantitative. Design: Linked administrative data (MUN postgraduate database; and Scott’s Medical Database) Location: Newfoundland, Canada Setting: Both Rural and Urban Physician focus: IMGs and CMGs.	• Community: Cultural and social isolation, • Professional: Limited professional mobility, fewer residency opportunities in preferred fields, and difficulty securing long-term employment.	• Personal: Familiarity with training location, • Professional: Integration into the healthcare system, and supportive practice environments. • Institutional: Residency matching process disadvantaging IMGs, lower likelihood of IMGs remaining in Canada or NL compared to MUN graduates, limited effectiveness of MUN residency programs in retaining IMGs.	• Enhance professional support, • Create structured career pathways, • Provide incentives beyond training and develop targeted retention policies for IMGs.
Mathews et al., 2008 [13].	To compare retention rates between provisionally licensed IMGs, fully licensed Memorial University Medical graduates (MMGs) and other fully licensed CMGs.	Study type: Quantitative. Design: Linked administrative data (Newfoundland College of Physicians and Surgeons; and Scott’s Medical Database 1997–2004) Location: Newfoundland, Canada Setting: Both Rural and Urban Physician focus: IMGs and CMGs.	• Personal: physician background and training (IMGs had lower retention rates although not different from CMGs) • Community: Social and cultural isolation in NL. • Professional: Not having a CCFP designation.	• Personal: MMGs retention rates were more due to ties with NL. • Community: Having cultural, religious or social ties to community. • Professional: Having a CCFP designation. • Local: NL was an entry point for most IMGs which increase recruitment but didn’t guarantee retention.	• Enhance cultural, social and professional supports. • Provide clear incentives and mobility options for IMGs. • Recruitment should focus on long-term retention goals rather than temporary workforce fixes.
Mathews et al., 2012 [36].	To examine generational differences in work location choices among physicians,	Study type: Qualitative Design: Interviews with physicians from different generations. Location: Newfoundland and Saskatchewan, Canada. Setting: Rural. Physician focus: CMGs only.	• Personal: Preference for urban settings with better career options and social opportunities. Work-life balance concerns, particularly among younger physicians who desire more flexible work conditions. • Family: Distance from family and social networks. Limited employment opportunities for spouses. Lack of quality education options for children • Community: Lack of community appreciation and engagement. Cultural and religious isolation. • Professional: Poor work environment. Limited access to medical resources, staff and updated equipment. High workload and burnout. • Systemic/institutional: lower remuneration. Billing restrictions and contract limitations. High physician turnover rates. Political instability and health policy changes especially from older generation physicians.	• Personal: Attraction to rural lifestyles particularly among those with prior exposure to rural life. Strong professional identity and commitment to patient care. • Family: available employment and education opportunities for spouse and children. • Community: community appreciation and engagement fostering a sense of belonging. Presence of cultural and religious networks. • Professional: positive collegial relationships and mentorships. Access to continuing medical education and career advancement opportunities. • Structural: Competitive remuneration and flexible incentive programs to make rural more attractive. • Improved administrative and policy support.	• Developing long-term retention strategies for physicians, particularly in rural areas. • Strengthening collegial and administrative support. • Increasing investment in medical infrastructure
Mathews et al., 2013 [40].	To examine and compare retention patterns among return-for-service (RFS) type physicians and non-RFS physicians	Study type: Quantitative Design: Administrative data (1997–2009) analysis on physicians with RFS and non-RFS agreements. Location: Newfoundland (NL), Canada. Setting: Rural. Physician focus: IMGs and CMGs.	• Family: Unavailability of suitable locations for family • Structural: Low bursary amounts, poor recruitment from sites, changes in personal priorities, lower completion rates for Special Funded RFS agreements, and lack of choice in accepting RFS agreements and work locations.	• Structural: Bursary-linked RFS agreements, allowing physician choice in work location, and alignment of individual preferences with provincial needs.	• Improve tracking to monitor RFS fulfillment of service obligations and implement penalties for defaulting • Alignment of provincial and federal RFS programs to maximize physician distribution in rural areas. • Encourage voluntary participation in RFS programs rather than enforcing RFS agreements with limited flexibility.
Mathews et al., 2017 [22].	To examine and compare the retention patterns of Canadians who study abroad (CSAs) and immigrant IMGs who completed post-graduate medical education (PGME) training in Canada.	Study type: Quantitative. Design: Registry data (National IMG Database and Scott’s Medical Database) Location: Canada (national scope). Setting: Both Rural and Urban Physician focus: Canadian IMGs or CSAs and immigrant IMGs.	• Personal: Gender (female IMG with a non-physician male spouse). • Community: lack of cultural networks in rural areas. • Professional: limited mobility in practice • Structural: Unclear return-of-service agreements.	• Personal: Gender (male IMGs with non-physician female spouse). • Community: Presence of cultural communities • Professional: Specialty type (family medicine vs. specialist). Family physician more likely to stay compared with specialist. • Structural: Eligibility for a full license	• Treating all IMGs equally in PGME selection, • improving IMG PGME training to enhance credentialing for independent practice, • Conducting further research to improve IMG performance and utilizing comprehensive data sets for informed policy-making and workforce planning.
Mathews et al., 2022 [35].	To explore the influence of residency match and ROS agreements on early-career decisions of IMGs.	Study type: Qualitative Design: Interview of early career IMGs Location: Canada (BC, Ontario, Nova Scotia). Setting: Rural Physician focus: IMGs	• Professional: Limited career choices, delayed preferred practice choices, and high turnover due to misalignment with location preferences. • Structural: Lack of autonomy in accepting ROS agreements, uncertainty about practice location and nature, inability to practice in preferred ways.	• Professional/Structural: Alignment of ROS requirements with personal and professional goals, such as desired practice location or family ties, facilitates retention.	• Revise ROS agreements to allow for flexibility with IMGs, • Provide flexibility in residency placements.
McDonald et al., 2012 [45].	To study the geographic mobility and retention of immigrant and non-immigrant physicians in Canada, with emphasis on the role of spousal characteristics.	Study type: Quantitative. Design: Geographic mobility analysis using Canadian Census data (1991–2006) Location: Canada (Provinces: Ontario, Quebec, Atlantic Provinces, Prairies, Alberta, British Columbia). Setting: Both Rural and Urban Physician focus: Both IMGs and CMGs	• Personal: Isolation in rural communities. • Family: Limited spousal integration in rural areas. • Community: High outmigration of physicians in rural areas making it more likely for new ones to migrate. Higher likelihood of outmigration among immigrant physicians.	• Personal: Proximity to urban amenities • Family: Spousal employment opportunities. Higher education level of spouse	• Improve spousal support systems. • Address isolation in rural areas. • Consider spousal employment in retention policies.
Mou et al., 2015 [32].	To explore determinants of inter-provincial migration intentions among rural and urban family physicians in Canada.	Study type: Quantitative Design: Analysis of National Physician Survey (2010) Location: Canada (Provinces: Newfoundland, Saskatchewan, Atlantic provinces, Ontario, British Columbia. Setting: Both Rural and Urban Physician focus: Both IMGs and CMGs	• Personal: Personal dissatisfaction with professional life and relationships. • Family: Spousal preferences and employment opportunities • Community: small population size and lack of urban-like amenities, language and communications barriers • Professional: Burnout from excessive on-call duties, and lack of time off for vacations or continuing medical education. • Systemic: insufficient impact of financial incentives and overhead cost of running clinics.	• Personal: Older aged, married physician. • Family: Spousal supports and opportunities for educational and professional advancements for spouse. • Community: Community appreciation, connection, active support, and physical/recreational assets; as well as larger community population size. • Professional: Collegiality among professional and social peer groups. • Systemic/organizational: Flexible primary care delivery models, favorable levels of compensation.	• Focusing on retaining IMGs, especially in rural areas. • Avoiding reliance on fee increases, addressing rural physician migration. • implementing flexible primary care models like Collaborative Emergency Centers (CEC) to enhance physician retention. • Implement strategies to mimic urban conditions such as spousal hire programs and professional development support
Mowat et al. 2017 [39].	To examine the retention and predictors of internationally trained family physicians in Manitoba.	Study type: Quantitative Design: Cohort study using Logistic regression. Location: Manitoba, Canada. Setting: Both Rural and Urban Physician focus: IMGs only.	• Family: Spousal employment, educational facilities for kids. • Community: Small community size and community disintegration. • Professional: Limited mobility due to conditional licenses, challenges in transitioning to remote areas, higher compensation and cultural diversity in other provinces (compared to Manitoba), and dissatisfaction with return-of-service contracts. • Systemic/Institutional: Removal of the Manitoba residency requirement before program entry, family relocation issues, the ineffectiveness of their mentorship program.	• Personal: Factors related to being established in Manitoba • Family: Spousal employment, appropriate educational facilities for children, • Community: Community integration both professionally and non-professionally, cultural diversity and access to cultural and religious institutions in community. • Professional: Licensure type and certification status with unlimited scope and mobility. Financial compensation. • Institutional: Manitoba residency at the time of application is a facilitator to retention, as it significantly increased the likelihood of IMGs remaining in Manitoba.	• Reinstating the requirement for applicants to reside in Manitoba before entering the program, • Exploring and potentially redesigning mentorship programs, • Considering factors beyond return-of-service contracts, such as community integration and family considerations, to enhance physician retention.
Myroniuk et al., 2016 [14].	To explore the influence of spouses on rural physician recruitment and retention.	Study type: Mixed Design: Surveys and interviews with physicians and their spouses, Location: Alberta, Canada. Setting: Rural Physician focus: Both CMGs and IMGs.	• Family: isolation, lack of job opportunities for spouses. Difficulty accessing higher education for spouse. • Community: Lack of social and cultural experiences. Cultural differences. Difficulty integrating into the community. Privacy/lack of anonymity concerns (for small areas) • Professional: Challenges with dual relationships in small communities.	• Personal: Proximity to work • Family: Supportive spouses, strong education system for children, spousal opportunities for employment, networking, and professional development • Community: Opportunities for cultural engagement, community support, safety, access to recreational activities, and community -directed retention efforts. Finding a good fit between personal and community culture • Professional: Collegiality, affiliation to academic center, broad scope of practice and specialist support. Personal and professional satisfaction. Continuing medical education (CME) support • Institutional: Greater academic and CME support.	• Academic and CME support, • Ensuring a good fit between physician skills and community needs, • Creating a centralized database of rural practice opportunities, • Enhancing the role of governing bodies in supporting physician spouses, and • Forming partnerships with private industry to support spousal employment.
Ogundeji et al., 2021 [37].	To explore the role of alternative payment models (APMs) in recruiting and retaining rural physicians.	Study type: Qualitative Design: Interviews Location: Alberta, Canada. Setting: Rural Physician focus: Both CMGs and IMGs	• Community: severe extremes in climate. Cultural and ideological differences, community pressures and lack of support • Professional: complex patient panel, limited access to specialist, poor work-life balance, and burnout • Structural: old and outdated equipment, lack of institutional support and access to specialist. Payment models/contracts that could lead to (a) potential to earn less; (b) fear of loss of autonomy and flexibility	• Personal: attraction to rural lifestyle, previous rural experience • Family: spousal employment, support and access education and childcare. • Professional: variety in scope of practice, good patient-physician relationship, collegiality, valued contribution to work, autonomy, independence, and financial compensation to stay. • Structural: relocation support and retirement plans	• Making APMs attractive through nonmonetary incentives, • Fostering a collaborative relationship between physicians and government, • Involving physicians in payment model design, ensuring fairness in workload and remuneration, and • Implementing accountability mechanisms to prevent perverse incentives.
Wasko, et al., 2014 [11].	To identify factors motivating physicians to select rural practice locations and remain in those communities.	Study type: Qualitative Design: interview based. Location: Saskatchewan, Canada. Setting: Both Rural and Urban Physician focus: Canadian Medical Graduates (CMGs) and IMGs	• Community: Community disintegration and discrimination. • Professional: Limited scope of practice, lack of independence and autonomy. Feeling unappreciated and alienated at work. • Institutional: Lack of financial incentives	Family: Physician and/or spouse having an attraction to rural lifestyle or having a rural background. Physicians and or spouse having ties to specific communities. Having friends and family living close to rural area. Spousal supports and opportunities for employment. • Professional: Broad scope and freedom of practice. Group practice. Positive work environment • Institutional: Adequate amount and mode of remuneration. Regional support (i.e., easy access to larger centers, specialist support. Having an attractive work schedule (e.g., with vacation time, locum relief, manageable call schedules, and opportunity for continuing medical education).	• Focusing on practice and lifestyle factors, • Improving work conditions such as more reasonable hours, better availability of locum tenens, and professional backup, • Providing educational opportunities for children, • Optimizing intra-professional collaboration, using technology to improve professional backup, and • Promoting the advantages of rural practice to younger physicians.
Witt, J. 2017 [41].	To investigate the opinions of physicians in Manitoba, Canada regarding rural jobs.	Study type: Quantitative Design: cross-sectional study. Location: Manitoba, Canada. Setting: Both Rural and Urban Physician focus: Canadian Medical Graduates (CMGs) and IMGs	• Professional: intense workloads, difficulty taking time off, professional isolation, lack of specialized education, lack of professional support, dissatisfaction with after-hours work, rural training, community incentives, clinic technology, inadequate work-life balance, and less preferred locations. • Structural: Financial incentives alone are insufficient for retention.	• Community: community incentives, • Professional: work-life balance, professional and social inclusion (e.g., group practice, • Structural: access to clinic technology), adequate housing availability, location preference (medium-sized towns within a 3-hour drive of Winnipeg), and appropriate financial compensation for on-call duties.	• Focusing on work-life balance, using nonpecuniary incentives such as professional and social inclusion (e.g., group practices, community incentives, access to telehealth), • Ensuring adequate housing availability and understanding the monetary value of compensating for undesirable job attributes. Financial incentives alone are insufficient for retention.

### Synthesis of findings

**Fig. 2:**
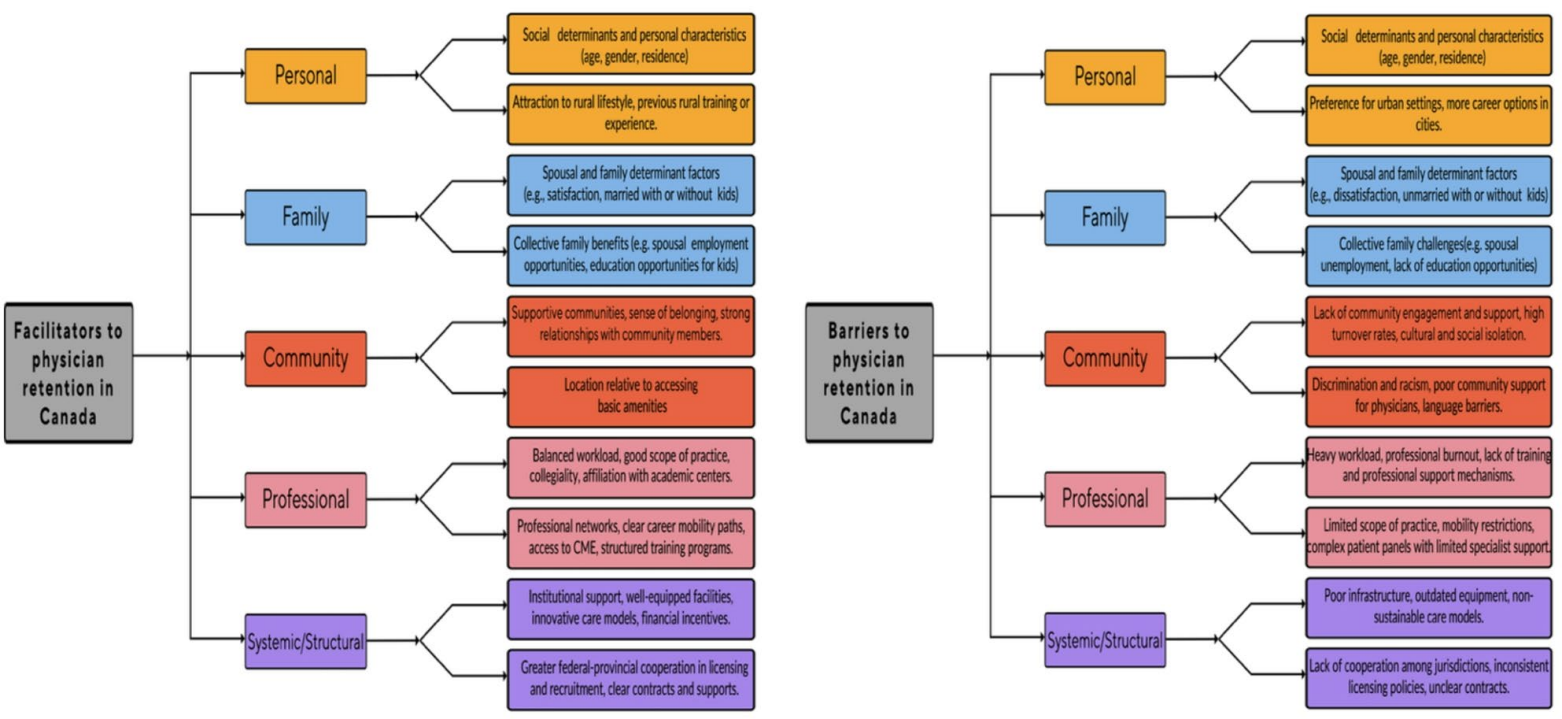
Summary of factors influencing physician retention in Canada

Thematic analyses across studies revealed retention factors which were mapped across the deductive coding framework of inter-related domains: personal, family, community, professional, and structural/systemic. Within each domain, both facilitators and barriers to physician retention were identified. A total of 16 major themes and over 30 sub-themes were classified and examined. See Table 5; Fig. 2, in which the findings from the synthesis were summarized.

**Table 5:** Factors influencing physician retention in Canada

A. **Barriers codified by domains**			
Factor	Theme	Description	Citation
Personal	Lifestyle preference	Preference for urban life, dissatisfaction with rural lifestyle	[22, 36, 44, 45]
	Isolation	Loneliness, lack of amenities, and distance from support networks	[36, 37, 43]
	Gender/relationship dynamics	Gender-related differences in spousal satisfaction and influence on location decisions	[22]
	Career satisfaction	Dissatisfaction with career path and/or misalignment with personal goals	[36, 38]
Family	Spousal employment	Lack of job opportunities for spouses in rural areas	[12, 14, 15, 32, 38]
	Children’s education and integration	Limited access to quality schools, educational disruption, social exclusion	[14, 39, 43]
Community	Social/cultural isolation	Lack of community engagement, cultural disconnect, absence of social networks	[14, 32, 36, 40]
	Discrimination	Experiences of racism, exclusion, or cultural insensitivity	[10, 32, 45]
	Community disintegration	Lack of amenities, poor infrastructure, fragmented support systems	[12, 15, 41, 45]
Professional	Burnout & workload	Excessive work hours, high call frequency, lack of relief or locum coverage	[22, 35, 38, 40, 43, 45]
	Limited mobility	Restricted license, return-of-service constraints	[22, 40]
	Restricted scope of practice	Unable to fully utilize training or practice independently	[10, 12, 15, 32]
	Lack of career development	Limited access to CME, mentorship, and opportunities for specialization	[36, 38]
Systemic	Licensing and policy constraints	Complex, inconsistent licensure across provinces; unclear ROS agreements	[32, 33, 44]
	Infrastructure and system instability	Underfunded health systems, unsustainable physician payment models, outdated facilities, lack of backup	[35, 39, 41, 43, 45]
	Administrative burden	Excessive non-clinical workload limiting satisfaction and efficiency	[38, 45]

B. Facilitators codified by domains			
Factor	Theme	Description	Citation
Personal	Lifestyle alignment	Alignment with rural values, preference for slower pace, love for natural environment	[13, 22, 33, 36, 38, 40, 43, 44]
	Sense of purpose	Feeling valued, having autonomy in care delivery, having meaningful patient relationships	[12, 13, 15, 22, 33, 36, 40, 44, 45]
Family	Spousal satisfaction	Spouses find meaningful work and feel welcome in community	[14, 32, 38]
	Children’s education and integration	Positive schooling options and social opportunities for children	[14, 39]
Community	Sense of belonging	Strong social connections, cultural fit, inclusion in community life	[12, 15, 32, 45]
	Community appreciation	Physicians feel respected and appreciated by community members	[12, 15, 35, 41]
Professional	Supportive team environment	Collegial relationships, collaborative practice, reduced isolation	[13, 43, 44]
	Full scope of practice	Opportunity to work to full potential with varied cases and independence	[17, 38, 45]
	Career development	Access to CME, professional mentorship, having academic affiliations	[18, 33, 35, 36, 38, 40, 43–45]
Systemic	Pan-Canadian licensure	Interprovincial mobility and flexibility in practice location	[18, 45]
	Flexible work arrangements	Blended payment models, locum supports, manageable workloads	[35, 39, 41, 43, 45]
	Residency and training alignment	Training in rural settings increases retention likelihood	[35, 36]

### Influences on retention

Our synthesis identified a range of interrelated barriers and facilitators across five domains: personal, family, community, professional, and structural/systemic. To enhance clarity and accessibility of findings, we structured this section to provide a domain-by-domain overview. Within each domain, common barriers and facilitators were highlighted, with particular attention to how these played out for urban/rural physicians and/or CMGs/IMGs, where relevant.

#### Personal domain

Barriers in the personal domain included lifestyle preferences (e.g., a desire for urban living among physicians placed in rural settings) and reluctance to relocate–especially among early-career physicians or those fulfilling RoS obligations without long-term intentions to remain [22, 35–37, 43–45]. Barriers in the personal domain included lifestyle mismatches, particularly for physicians recruited to rural areas who preferred urban amenities or social environments. IMGs, especially women, were more likely to report dissatisfaction when their personal and professional identities clashed with recruitment expectations. Facilitators on the flip side, included personal alignment with rural life, a strong sense of calling or mission, and feeling valued in one’s role.

#### Family domain

Family-related barriers were prominent across most included studies. The inability of spouses to find meaningful employment, and concerns over children’s education and social integration, were recurrent themes across CMGs and IMGs in both urban and rural settings [14, 32, 38, 39]. However, these challenges were particularly salient for IMGs, who often lacked extended family or support networks. Conversely, when spouses were employed and children integrated well, retention improved [14, 32, 38, 39].

#### Community domain

A sense of social and cultural isolation (particularly among racialized physicians) emerged from our synthesis as a significant barrier to retention [10, 12, 14, 15, 36, 40, 41, 45]. Rural practitioners (and a few urban-based physicians) commonly cited the absence of amenities that contribute to quality of life (such as gyms, parks, and community centres) as well as poor infrastructure, including under-resourced hospitals, clinics, Emergency Departments, diagnostic services, and unreliable tele-health systems. Many also reported experiences of discrimination and disconnection from the social fabric of the community. Conversely, strong community relationships, cultural belonging, and visible appreciation were noted as powerful enablers of retention [12, 15, 32, 41, 45].

#### Professional domain

The most cited barriers were burnout, unsustainable workloads, limited scope of practice, and lack of mentorship–which cross-cut urban and rural family physicians [12, 15, 22, 33, 35, 36, 38, 40, 43, 44]. However, for rural physicians, these were exacerbated by the extra call burdens and added professional isolation [12, 15, 22]. While rural practices were often assumed to offer broad scope opportunities, some studies [12, 33, 35, 38, 40, 42–45] revealed that constraints (such as restrictive billing codes, limited team supports, or administrative oversight) narrowed practice scope, leaving physicians underutilized or unsupported in delivering comprehensive care. For IMGs especially, the challenge was not the scope itself, but the lack of mentorship and sustainable infrastructure to manage their scope of practice effectively. Conversely, facilitators in this domain included collegial teams, full-scope autonomy, and embedded support networks such as local CME, mentorship, and academic affiliations [22, 33, 35, 36, 38, 40, 41, 43, 44].

#### Structural/Systemic domain

Systemic challenges included restrictive licensure (particularly for IMGs), unclear and sometimes punitive RoS contracts, and under-resourced clinical settings (common in rural practices). Additionally, systemic barriers disproportionately affected IMGs and rural physicians [17, 18, 22, 34–36, 45, 46]. Systemic facilitators however, included streamlined licensing, flexible payment models (e.g., alternate payment models), and well-funded infrastructure that supported interdisciplinary care delivery [18, 32, 35, 36, 45].

Themes and citations for each domain are summarized in Table 5 and illustrated in Fig. 2.

### Persona-based synthesis

The identification of personas help visualize how the cross-cutting themes and domain-level factors manifest in real-world decision-making scenarios, especially for rural and IMG physicians. They bring to life how context shapes whether barriers are overcome or compounded. A series of seven conceptual physician personas were developed based on recurring patterns across included studies and stakeholder validation sessions. Each persona represents a composite of lived experiences, illustrating how combinations of personal, family, professional, community, and structural influences shape decisions to stay or leave family practice in Canada. For example:

- *The Frustrated IMG* represents an early to mid-career internationally trained physician (ITP) navigating restrictive licensing pathways, family integration challenges, and community isolation, often with spousal dissatisfaction compounding the risk of attrition.
- *The Burned-Out Rural Physician* represents a committed mid to late-career rural FP overwhelmed by unsustainable workloads and lack of relief or support, particularly from emergency and hospital systems.
- *The Rural-Rooted Generalist* represents a Canadian-trained physician aligned with rural life and comprehensive care values, but is vulnerable to burnout if institutional, professional or peer support, adequate CME, or mentorship are lacking.
- *The Mobile Urbanist* represents a lifestyle-driven early-career physician preferring urban amenities and flexible practice; unlikely to remain in rigid or geographically isolated settings.
- *The Early-Career Explorer* represents a new resident graduate unsure of long-term goals; responsive to mentorship, manageable workloads, and opportunities to try varied models of care.
- *The Trusted Community Doctor* represents a mid to late-career physician deeply integrated into their community and driven by service and continuity but may be pushed out by mounting burnout or lack of systemic support.
- *The Dual-Career Physician* juggles personal and partner career demands, particularly in two-physician or professional households, requiring location flexibility and spousal support to remain.

**Fig. 3:**
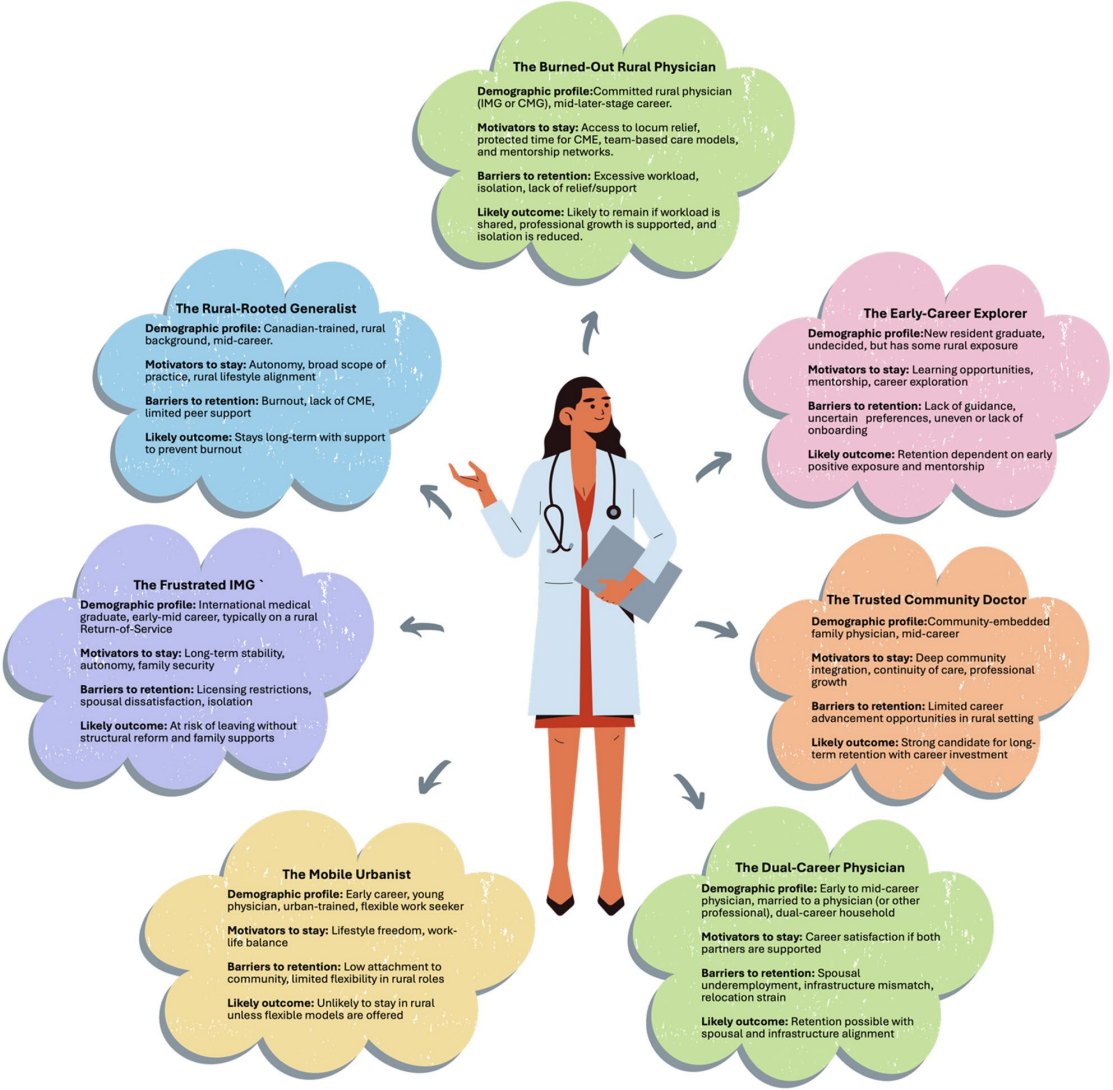
Conceptual physician personas constructed from the study

These personas are not meant to be exhaustively representative as they only provide a narrative lens to begin to understand how cross-domain interactions (e.g. spousal dissatisfaction combined with professional stagnation) compounded retention risks. They could potentially serve as tools for policymakers to tailor solutions that aligned with the unique needs of different physician profiles. (see Fig. 3).

### Recommendations for retention

We identified a set of evidence-informed recommendations mapped to the five domains. These included the need to streamline licensure processes across provinces [18, 32, 38], revise RoS agreements for better alignment with physician goals [40, 44], strengthen spousal and family support systems [14, 38, 39], invest in community-based onboarding and cultural inclusion programs [10, 12, 15, 32, 45]; as well as develop flexible, team-based models of care [17, 35, 45].

While it is possible that pan-Canadian licensure could increase physician mobility between provinces, our review findings suggested that simplifying licensure requirements may also encourage retention by reducing administrative burden (e.g., redundant paperwork, fees, background checks and delays), enhancing professional autonomy (delimiting physicians’ ability to respond flexibly to evolving career goals, personal needs and short term relocations), and improving access to short-term locum or cross-provincial practice opportunities. For many early-career physicians, particularly IMGs, the ability to engage in temporary practice outside their home province without reapplying for licensure offered a sense of flexibility and control that supported long-term engagement in their primary practice location [18, 32, 38]. As such, rather than driving attrition, streamlining licensure was viewed as a way to reduce that frustration by enabling an environment for professional growth, and support, to remain in underserved areas while staying connected to broader networks of care. See Table 6 for more description of identified recommendations.

**Table 6:** Recommendations to enhance physician retention in Canada (codified by domains)

Factor	Theme	Description	Citation
Personal	Align with values and lifestyle	Recruit and retain physicians whose values align with rural practice (e.g., autonomy, slower pace, community-focused care)	[13, 33, 36, 40, 43]
Family	Support for spouses and children	Develop dual-career recruitment policies, offer family integration supports, and invest in schooling and social amenities	[14, 38, 39]
Community	Community onboarding and inclusion	Develop welcoming strategies, cultural safety training, and long-term engagement efforts for physicians and their families	[12, 15, 32, 45]
Professional	Mentorship and career development	Strengthen CME access, mentorship networks, academic affiliations, and rural teaching opportunities	[36, 38, 43]
	Team-based and flexible care models	Implement collaborative practices, group models, and reduce isolation through peer and specialist support. Payment models should support interdisciplinary teams and compensate for non-clinical activities (e.g., case conferencing, care coordination).	[17, 35, 45]
Systemic	Pan-Canadian licensure and mobility	Adopt national licensing models to reduce barriers and enhance locum support and career flexibility	[18, 32, 38]
	Sustainable RoS and payment models	Co-design RoS agreements that align with physicians’ goals and local community needs. Implement alternate (e.g., blended or capitation) payment models that reward continuity, support flexibility, and reduce the volume-driven pressures of fee-for-service. These payment models should be embedded within broader strategies that support collegial environments, family integration, and community belonging.	[17, 18, 32, 35, 36, 40, 44, 45]
	Data-informed planning and evaluation	Monitor long-term retention trends, especially among IMGs. Link data on physician workload, billing, and outcomes to inform payment reforms aligned with retention goals.	[14, 44, 45]
	Reduce administrative burden	Cut non-clinical workload and streamline documentation to improve physician satisfaction and efficiency	[15, 38]

## Discussion

Retention of family physicians in Canada is more than a workforce issue; it is a lens through which we can see the strengths and cracks in our healthcare system. The decision to stay or leave, particularly in Family Medicine, is never made in a vacuum. It is often a decision shaped by the delicate interplay of personal aspirations, systemic conditions, community dynamics, and the professional eco-systems that family physicians find themselves in [47]. Our integrative review draws together 25 years of research to illuminate the real-world conditions that influence the decision of family physicians to stay or leave practice in Canada.

While literature on health workforce sustainability frequently highlights common barriers such as burnout, licensure restrictions, spousal dissatisfaction, community integration, and more, few studies have synthesized these factors in ways that capture their cumulative or interacting effects. In mapping and integrating our study findings across five interconnected domains (i.e., personal, family, community, professional, and structural/systemic), we showed how mis-alignment across any of these spheres can break a family physicians’ sense of purpose, belonging, or sustainability [7, 48–50].

As such, our findings showed that physician retention is deeply relational; as physicians tended to stay where they felt connected, whether to communities, colleagues, a sense of purpose, or systems that valued their work. Conversely, departures were often driven by the erosion of these relationships. The patterns of family physicians that stay versus those that leave practice were also corroborated by healthcare providers (family physicians and NPs) in our World Café study [51] which pointed out how deep relationships influenced their decision to stay or leave (and this was especially true for physicians working within rural or remote areas in Saskatchewan). In such locations, the departure of even one physician could collapse a clinic, destabilize a regional area, and leave patients with no option but to travel hours to receive primary care [10, 16, 51]. These communities (often home to Indigenous and underserved populations) bear the compounded effects of underinvestment, systemic neglect, and colonial legacies [52]. In such contexts, retention is not just about staffing but also about relational equity: where rural healthcare providers face heavier clinical burdens, fewer specialist supports, and greater isolation than their urban peers [18, 32, 40, 53–56]. Physician frustration in these settings, as our synthesis and World Café study revealed, stemmed not from a lack of commitment, but from the absence of conditions required to thrive [51]. Retention, then, should be reframed not only as a workforce strategy but also as an issue of equity [57, 58], fairness [59], reconciliation [59–61], and investment in structurally excluded communities [62, 63].

One of the unique contributions of this review was the development of physician “personas” (i.e., conceptual profiles grounded in the synthesis of retention narratives across the included studies). These personas (ranging from the “*Frustrated IMG*” facing the *usual* licensing hurdles and family isolation, to the “*Burned-Out Rural Physician*” managing years of practice with vacillating support) brought humanity into the discussion. Often times, the discussion around physician retention, tends to create an abstract picture or caricature of the typical family physician in Canada. It is believed that the physician “personas” are not caricatures but composite portraits of “real” and lived physician experiences that reveal the emotional, and identity-based dynamics that shape physician life, family, and career trajectories [38, 45, 47, 57, 62, 64–66]. More importantly, these personas also revealed how generic, one-size-fits-all policies have often reinforced (rather than alleviated) the challenges faced by physicians across Canada. Thus, we can now appreciate that a one-size-fits-all approach for retention cannot address the divergent realities of a young CMG parent seeking urban flexibility, or an IMG navigating integration challenges, or a mid-career rural physician on the verge of exhaustion and so on. Each of these physicians needs something different. On the other hand, it cannot be assumed that the personae framework offers a perfect solution in itself; we believe it proposes a way to think more carefully, more empathetically, and more practically about what different family physicians need in order to stay in their home province or in Canada to practice. It is believed that our study findings may assist decision-makers to shift from the *usual* reactive workforce planning to an *intentional* design of retention in Canada (and by extension similar primary care systems globally). For example, a well-functioning retention ecosystem, might include *bundled* supports such as flexible licensure pathways across provinces, locally tailored onboarding and peer mentorship programs, spousal employment supports, access to locum coverage, and embedded community engagement efforts, each calibrated to the physician’s career stage and practice context (opposed to a generic often lobsided strategy).

Building on this foundation, our study also revealed how deeply interconnected the factors influencing physician retention are across personal, family, community, professional, and structural domains. For example, structural constraints (such as restrictive licensure pathways, rigid return-of-service contracts, and traditional fee-for-service (FFS) payment models) often collide with professional challenges like lack of autonomy, limited team supports, and excessive administrative workload. Among these, the FFS model emerged as a consistent and negative influencing factor to retention because it rewards volume over continuity, undervalues relational and preventive care, and contributes to burnout through time pressure and documentation load.

Our synthesis showed that reimagined/remodelled payment reform could act as both a structural and relational lever for retention. Our synthesis showed that blended or capitation models were associated with improved job satisfaction and lower turnover/attrition by: allowing physicians to organize work around patient needs rather than billing constraints [32, 67]; enabling interdisciplinary collaboration by funding non-clinical coordination time [17, 38, 45, 46, 67, 68]; stabilizing income while reducing dependence on long clinical hours [38, 45, 67, 68]; and rewarding long-term patient relationships and ongoing practice stability [17, 43, 69]. However, remuneration reforms alone may be insufficient as it could create unintended consequences such as misaligned incentives and inequitable resource distribution [2, 70–72]. The impact of remuneration reform on retention has been shown to be amplified when integrated with complementary strategies such as team-based care models, mentorship and peer networks, spousal and family supports, and community onboarding [67–69]. In essence, payment models set the structural conditions for sustainability, but the relational and professional environment determines whether physicians thrive within those structures (69). Therefore, retention strategies should view remuneration reform not as an isolated fix, but as part of a coordinated ecosystem that aligns financial incentives with physician well-being, community belonging, and continuity of care.

Alas, the consequences of less-than-optimal physician retention in Canada threatens the very viability of Family Medicine as a discipline [19, 73]. For example, fewer medical students are choosing Family Medicine as a career [66, 74–76]. Furthermore, early-career family physicians are gravitating towards more focused practices that allow for more control and flexibility [77–79]. Comprehensive, community-based generalist care is increasingly perceived as unrewarded and unsustainable [79–82]. This unfortunate trajectory undermines the core principles of the Patient’s Medical Home [83, 84], and the broader vision of universal, relational primary care that Canada has long been espoused [83–85]. Without a course correction, we risk creating a healthcare system in which continuity is the exception rather than the norm [73, 86]. However, there are glimmers of hope and reform for retention strategies, such as the expansion of interdisciplinary teams [83, 84, 86, 87], the re-design of alternate payment models [38, 73], the strengthening of IMG assessment pathways (including the CMA’s proposal for a Pan-Canadian licensure pathway) [22, 88, 89], and the emergence of wellness and mentorship initiatives aimed at supporting physicians [89, 90]. Notably, the CFPC’s Patient’s Medical Home (PMH) Model offers a compelling vision for team-based, community-grounded care [83, 84]. In addition, the Canadian Medical Association’s advocacy for Pan-Canadian licensure proposes regulatory reforms to reduce interprovincial barriers to practice [88]. Province-specific strides are being made, which can be used as models for optimizing primary care delivery across the country. In Nova Scotia, expanded team-based care and centralized physician recruitment have been implemented [91]. British Columbia introduced a new longitudinal payment model to support comprehensive care [92]. Alberta is modernizing its Primary Care Networks to facilitate primary care team attachment and access [93]. Saskatchewan recently rolled out a new remuneration model to strengthen physician retention in urban, rural and remote communities [94], has ongoing participation in provincially-funded primary care innovation specific to individual clinic and community needs, and is working to re-align primary care delivery among networks [94]. Manitoba has rolled out rural mentorship and retention bonuses for primary care teams and the health workforce [95], and Newfoundland and Labrador are developing Family Care Teams alongside incentive structures [90]. These provincial efforts represent important steps toward a sustainable primary care workforce. While these reforms are an important step forward, we believe they should be co-created *with*–and not *imposed on*–the relevant stakeholders (e.g., physicians, nurses, patients, other regulated health practitioners, Indigenous communities, and decision-makers), adapted to local contexts, and evaluated rigorously to ensure long-term sustainability.

Ultimately, our integrative review offers a people-centered framework for understanding and enhancing physician retention in Canada. Physicians remain in practice when they feel seen, supported, and aligned with the communities they serve. Retention, in this light, is not merely about filling positions but about building systems in which care relationships, and thereby care delivery, can thrive.

### Strengths and limitations

Our review offers a comprehensive and nuanced understanding of family physician retention in Canada, drawing upon 25 years of empirical literature across diverse geographic, demographic, and practice contexts. One of the key strengths of this study is its use of an integrative approach, which enabled the synthesis of qualitative, quantitative, and mixed-methods research to identify patterns, tensions, and intersections that may not have been visible through a single-method lens. The development of physician “personas” (i.e., conceptual profiles grounded in real-world narratives) further distinguishes this review by humanizing retention trends and making them more accessible to decision-makers, educators, and policy leaders. Another strength lies in the focus on relational and contextual drivers of retention across five intersecting domains: structural, professional, community, family, and personal. This approach moves beyond reductionist or transactional explanations and offers a systems-level perspective that may inform more responsive, tailored, and equity-oriented retention strategies.

However, several limitations should be acknowledged. First, while reflections on policy gaps were included in our analysis, this study did not involve a formal review or synthesis of Canadian policy documents. As such, policy-related insights should be interpreted as themes derived from the included literature, rather than as conclusions from a comprehensive policy analysis. Second, while our inclusion criteria were intentionally broad to capture diverse physician experiences, certain regions (e.g., Canada’s northern territories) were under-represented in the data, limiting generalizability to those contexts. Third, most included studies did not disaggregate findings by physician identities (e.g., racialized groups, gender-diverse individuals), which may mask intersectional experiences of retention or attrition. Regardless, we believe this review contributes meaningfully to ongoing conversations about health workforce sustainability by offering practical, person-centered insights that can guide future practice and policies.

## Conclusion

In conclusion, through this integrative review, we have identified a range of inter-related factors that influence the retention of family physicians in Canada across five domains: structural/systemic, professional, family-related, community, and personal. Structural and professional barriers such as administrative burden, restrictive licensure, and limited autonomy, emerged as the most significant roadblocks to retention. Facilitators were often grounded in family and community support, collegial work environments, and a sense of belonging. By synthesizing findings across 23 studies and mapping them to conceptual physician personas, this review underscored the importance of co-creating tailored and context-specific physician retention strategies, including, but not limited to, reforming remuneration models, implementing team-based primary care, creating Pan-Canadian licensure pathways, and enhancing community-driven integration efforts. Overall, this review provides actionable insights for healthcare practitioners, policymakers and health system leaders that would strengthen sustainability of the primary care workforce in Canada.

## Supplementary Information

Supplementary Material 1.

## Data Availability

The datasets used and/or analysed during the current study are available from the corresponding author on reasonable request.

## Abbreviations

APMs: Alternate Payment Models
CCFP: Certification in the College of Family Physicians
CIHI: Canadian Institute for Health Information
CMG: Canadian Medical Graduate
CME: Continuing Medical Education
CSA: Canadian Studied Abroad
CFPC: College of Family Physicians of Canada
CMA: Canadian Medical Association
ERIC: Education Resources Information Center
IMG: International Medical Graduate
JBI: Joanna Briggs Institute
LFP: Longitudinal Family Physician
MMG: Memorial University Medical Graduate
MUN: Memorial University of Newfoundland
NPS: National Physician Survey
PMH: Patient’s Medical Home
PGME: Postgraduate Medical Education
PRISMA-ScR: Preferred Reporting Items for Systematic Reviews and Meta-Analyses Extension for Scoping Reviews
RoS: Return-of-Service
SIPPA: Saskatchewan International Physician Practice Assessment
SRPC: Society of Rural Physicians of Canada
UoS: University of Saskatchewan
